# The complete mitochondrial genome of *Aparapotamon similium* (Decapoda: Brachyura), an endemic to China

**DOI:** 10.1080/23802359.2019.1638323

**Published:** 2019-07-13

**Authors:** Huimeng Liu, Fengjuan Jiang, Shuxin Xu, Chunchao Zhu, Xianmin Zhou, Jiexin Zou

**Affiliations:** a Research lab of Freshwater Crustacean Decapoda and Paragonimus, School of Basic Medical Sciences, Nanchang University, Nanchang, Jiangxi, PR China;; b Key Laboratory of Poyang Lake Environment and Resource Utilization, Ministry of Education, Nanchang University, Nanchang, Jiangxi, PR China

**Keywords:** Complete mitochondrial genome, *Aparapotamon similium*, phylogenetic analysis

## Abstract

In this study, we first obtained the complete mitochondrial genome of *Aparapoamon similium* (Decapoda: Brachyura). The complete mitochondrial genome is 19,236 bp in length and includes 37 typical genes (13 protein-coding genes, 22 tRNAs genes, 2 rRNAs genes, and 1 putative control region). The whole mitochondrial genome is characterized by the apparent AT bias (72.82%). BI and ML phylogenetic analysis based on 67 mitochondrial genomes of Brachyura species show a highly similar topology structure with high bootstrap supported. The results reveal the close relationship between *A. similium* and *Potamiscus motuoense.* This study would establish a solid data foundation for further diversification studies.

Among extant crustaceans, brachyuran crabs, containing more than 7250 described species, are considered as one of the most species-rich clades (Davie et al. 2015). However, the molecular data in public database is still scarce. It has been reported that complete mitochondrial genomes contain enough information to reconstruct phylogenetic relationship (Dabney et al. 2013; Guschanski et al. 2013). In this study, we first determine the complete mitochondrial genome of *A. similium*, describe its genome level characteristics and try to figure out its phylogenetic status.

*Aparapotamon similium* belongs to Arthropoda, Crustacea, Malacostraca, Decapoda Apatapotamon, and Potamoidea. It is endemic in Yunnan Province, China. Differing from other genus, *A. similium* is recognized as typical Alpine type inhabiting in mountains above 1500 meters (Dai 1999). We collected an individual in Ninglang County (26.96°N and 100.96°E), Lijiang City, Yunnan Province, China at the altitude of 2766 meters in 2017. Then, the sample was well preserved in 100% ethanol and stored at 4°C until DNA extraction. The whole experimental protocols were referred to as the ways routinely used before (Jia et al. 2018).

The complete genome of *A. similium* is 19,236 bp in length (GenBank accession number: MK950854) and contains 13 protein-coding genes (PCGs), 22 tRNA genes, 2 rRNA genes, and 1 non-coding control region (D-loop). The base composition of the whole genome shows high A + T content (70.59%). A total of non-coding gene is 4,524 bp in length, which contains 21 non-coding regions, ranging from 1 to 297 bp. Nine gene overlaps of 1–7 bp in length are observed and the longest one are located between ND4 and ND4L. Four of the 13 PCGs are encoded by the L-strand, and the other genes are encoded by the H-strand. Six of the 13 PCGs (COX1, COX2, COX3, ATP8, ND4L, and ND5) use ATG as the start codon, three genes (ATP6, ND1, and ND2) start with ATA, three genes (ND3, ND6, and CYTB) treat ATT as the start codon and ND4 use GTG as the start codon. In addition to ND3 use TAG as the stop codon, CYTB and ND5 have incomplete stop codon with T– and the remaining 10 genes end with TAA. The 22 tRNAs are 61 bp (tRNAArg) to 72 bp (tRNAVal) in length. All the tRNAs can form typical cloverleaf structure, except for tRNASer (AGN), which is common in metazoans (Ohtsuki et al. 2002). Eight of the tRNA genes are encoded by the L-strand and the rest of the genes are encoded by the H-strand. The 16S and 12S rRNA genes are located between tRNALeu (UUR) and tRNAVal, respectively, separated by tRNAGln gene. The control region (CR) of *A. similium* exists between 12S rRNA and tRNAIle genes, is 1664 bp in length and has an AT content of 81.73%.

ML and BI phylogenetic trees are performed using mitochondrial genome sequences of 67 Brachyurans (including *A. similiuma*) with 13 PCGs and choose *Clibanarius infraspinatus* as an outgroup. The results exhibit highly similar topology structure, which presents high bootstrap support (Figure 1). The phylogenetic trees uncover the sister position between *A. similiuma* and *Potamiscus motuoense* which is derived within the freshwater crabs clade and it is sister to *Somanniathelphusa boyangensis*. Thus, according to the phylogenetic result, freshwater crabs have clustered together. Meanwhile, *A. similiuma* and *Potamiscus motuoense* have a close genetic relationship.

**Figure 1.**
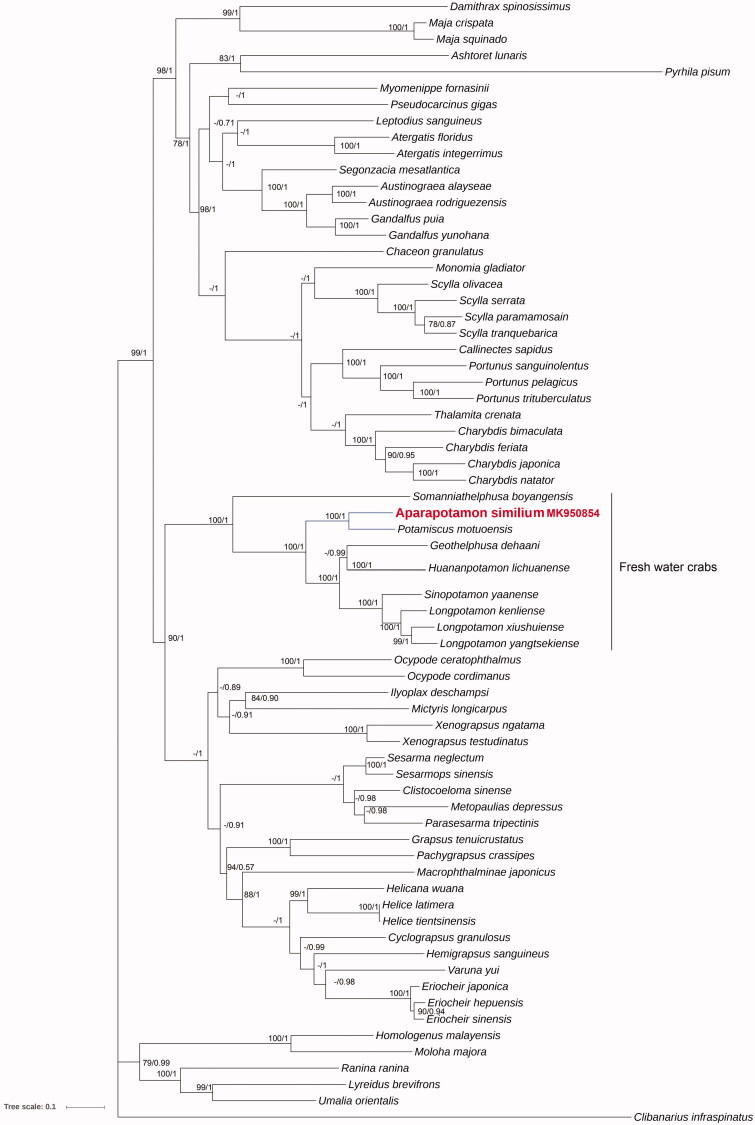
Phylogenetic Bayesian inference (BI) tree of *A. similiuma* and related brachyurans based on 13 PCGs sequences from the mitochondrial genome; *Clibanarius infraspinatus* serves as the outgroup. The numbers at the internodes are Bayesian inference (BI) bootstrap proportions and maximum likelihood (ML) posterior proportions. The differences between the ML and BI trees are indicated by ‘–’. The scale bars represent genetic distance.
